# Management of the Cavity After Removal of Giant Cell Tumor of the Bone

**DOI:** 10.3389/fsurg.2021.626272

**Published:** 2021-07-29

**Authors:** Yushan Wang, Qiaoqiao Tian, Chenyang Wu, Haoze Li, Jian Li, Yi Feng

**Affiliations:** ^1^Orthopedics Department, Second Hospital of Shanxi Medical University, Taiyuan, China; ^2^Department of Computer & Information Technology, Shanxi University, Taiyuan, China

**Keywords:** giant cell tumor of bone, surgical treatment, local control, filling materials, osteogenic induction

## Abstract

**Purpose:** To find out the most appropriate management scheme through the analysis and comparison of different inactivation methods and filling materials.

**Method:** A systematic literature search was performed using the terms, anhydrous ethanol, phenol, hypertonic saline, cryotherapy, thermal therapy, bone reconstruction, GCTB, and etc., Selected articles were studied and summarized. The mechanism, clinical effects, and influence on bone repair of various methods are presented. Recent developments and perspectives are also demonstrated.

**Recent Findings:** Compared to curettage alone, management of the residual cavity can effectively reduce the recurrence of giant cell tumours of bone. It is a complex and multidisciplinary process that includes three steps: local control, cavity filling, and osteogenic induction. In terms of local control, High-speed burring can enlarge the area of curettage but may cause the spread and planting of tumour tissues. Among the inactivation methods, Anhydrous ethanol, and hyperthermia therapy are relatively safe and efficient. The combination of the two may achieve a better inactivation effect. When inactivating the cavity, we need to adjust the approach according to the invasion of the tumour. Filling materials and bone repair should also be considered in management.

## Introduction

**Figure 1:**
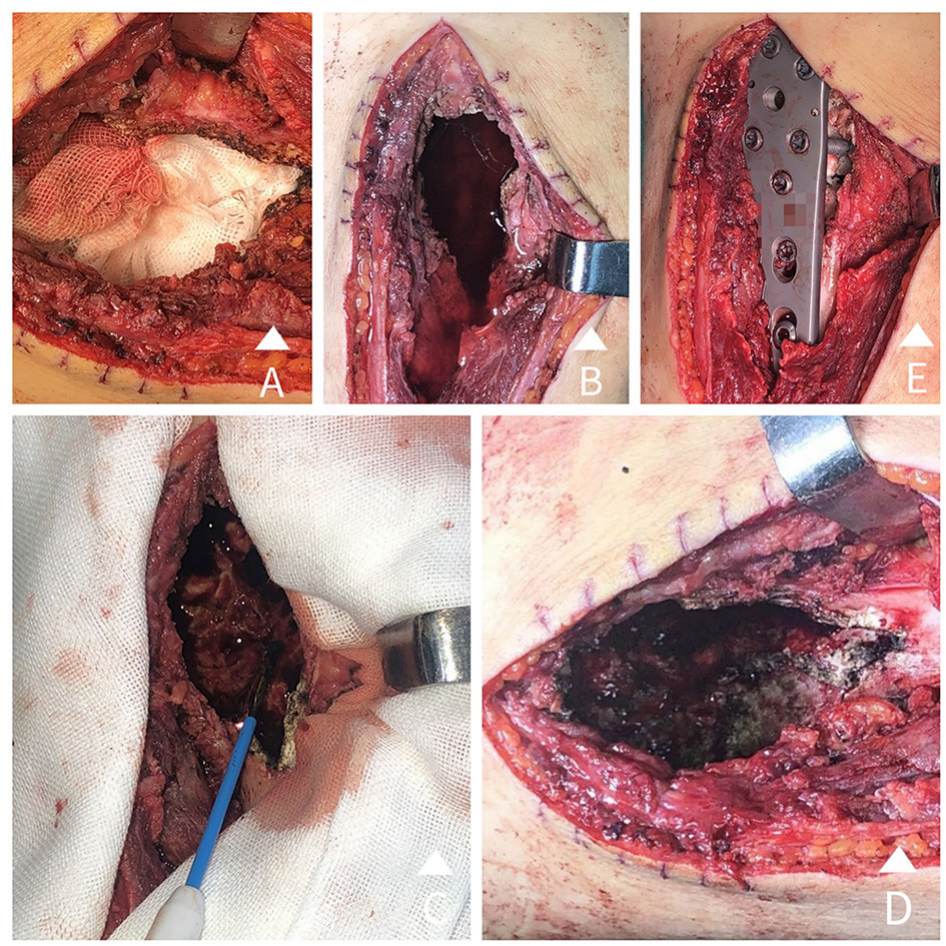
**(A)** A large incision, a large bone window opening, and tumour curettage are conducted. **(B)** Anhydrous ethanol is poured into the tumour residual cavity and set for 15 minutes. **(C)** The residual cavity wall is heated with electrotome thermocoagulation inch-by-inch under proper protection. **(D)** The residual cavity wall is obviously carbonized after heating. **(E)** Internal fixation is carried out using nails and steel plates after closing the bone window.

**Figure 2:**
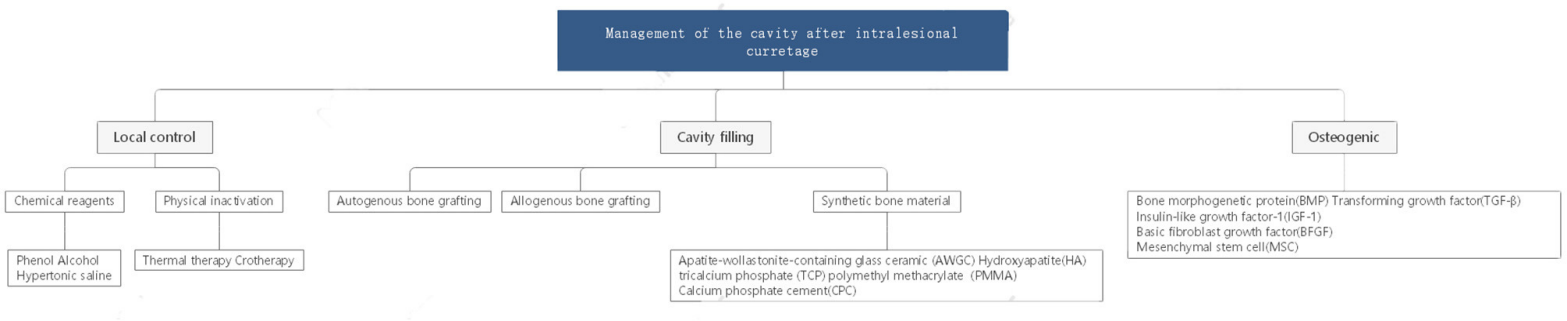
Management of the cavity after intralesional curretage.

Giant cell tumour of bone (GCTB) is one of the most common primary bone tumours, and its incidence in China is as high as 14.9%. Treatment of this tumour has been controversial in recent years because GCTB is more aggressive than normal benign tumours. However, extensive intralesional curettage is still the most commonly accepted procedure. This surgical method can not only ensure postoperative function but also reduce the recurrence and metastasis rate to a certain extent. This method is also widely used for other benign lesions that are prone to relapse and malignant transformation, such as enchondroma, aneurysmal bone cyst, and chondromyxoid fibroma. Operationally, the surgeon should first open a bone window sufficiently large to manage the tumour and residual cavity under direct vision and then perform curettage, inactivation, and reconstruction (Figure 1). During these steps, management of the residual cavity is as important as complete curettage to reduce postoperative recurrence. Studies have shown that the recurrence rate after simple curettage is relatively high, ranging from 25 to 50% (1–6). Curettage combined with residual cavity management can effectively reduce the recurrence rate (1, 7, 8). Currently, there are three vital pillars for management of the tumour cavity (Figure 2):

1. Local control. Local tumour control and extensive clearance are crucial for decreasing the existing microlesions and avoiding further invasion and recurrence.
2. Cavity filling. Cavity filling and hard material implants are effective in promoting early functional recovery and mechanical stability.
3. Osteogenic induction. Inhibiting fibrous proliferation and promoting osteogenic differentiation are major steps in tumour cavity management to accelerate self-reconstruction and incorporation of filling materials.

Almost all studies on the treatment of bone defects in GCTB are based on the three theories mentioned above. To date, many methods have been tested. These methods are reviewed in this article. Trends and updates regarding GCTB management are also introduced.

## Methods

We searched Medline, EMBASE, and PubMed with the terms GCTB, intralesional curettage, adjuvant therapy, high-speed burring, denosumab, juxta-articular giant cell tumour of bone, bone defect, bone repair, thermoablation, cryotherapy, phenol, ethanol, and bone graft. After a cursory review of more than 200 studies, we read 100 of them thoroughly. The inclusion criteria were as follows: (1) literature published mainly between 2005 and 2020; (2) all clinical retrospective studies of curettage combined with adjuvant therapy for the treatment of GCTB; (3) all reviews of methods on residual cavity inactivation; (4) literature on the repair of residual bone defects after adjuvant therapy; and (5) basic studies on tolerance of bone repair-related factors in a special environment. The following articles were excluded: (1) articles on segmental resection and drug therapy in GCTB; (2) case reports and (3) articles on adjuvant therapy combined with radiation and chemotherapy. Majority of my information was derived mainly from articles in the last 10 years. The key focus of this review is to understand various methods of residual cavity management from various aspects. Different methods are compared, and their advantages and disadvantages are summarized. Finally, recommendations should be made for clinicians to treat GCTB more effectively. Majority of my information was dervied mainly from articles in the last 10 years.

## Review and Findings

### Tumour Cell Elimination

Sufficient curettage and a second elimination using adjuvant agents or physiotherapy are needed in GCTB. There are data indicating that when local adjuvants are not utilized, the mean recurrence rate is ~42% (21–65%) (9–16). In contrast, recurrence rates of less than 20% have been cited in the literature following the addition of adjuvant therapies to curettage (13, 17–19). In the following section, we describe and compare various inactivation methods from the perspectives of the principle, application, range of influence, clinical effect and influence on bone repair.

#### Chemical Reagents

##### Phenol

Phenol is an important reagent that has been used for a long time. Until approximately 2010, one of the most established standard treatments with acceptable recurrence rates was curettage with local adjuvant application of phenol and polymethyl methacrylate (PMMA) (recurrence rate, 3–33%) (12, 13, 15, 16, 20–26). Phenol is a chemical agent that induces tumour necrosis and coagulation of proteins (27, 28). The concentration of phenol for local inactivation ranges from 5 to 95%. The effects achieved by using 5% phenol are safer for patients (29, 30). Increasing the temperature does not significantly increase the therapeutic effect but does increase the absorption of phenol and lead to poisoning (31). Usage includes a direct infusion or smearing the wall of the residual cavity using a tampon or gauze. However, we think the effect of soaking is much better than that of surface coating. To achieve the ideal therapeutic effect, phenol should be applied at least 3 times. Phenol application times ranging from 1 to 6 min have been reported in different studies. Studies have shown that it takes 6 min for phenol to effectively kill GCT cells (32). The cytotoxic effect of phenol has been studied *in vitro* on monolayer cultures of cells from GCTB, and the infiltration depth has been estimated at 0.2 mm (33). The *in vivo* effect was studied in fresh animal cadavers (34), and in 90% of cases, phenol caused a mean 566-μm-wide zone of devitalization of bone marrow cells. To date, there have been no direct *in vivo* experiments in humans. Therefore, the real infiltration depth and specific reaction mechanism are still unknown, especially for different locations. We hypothesize that tissues adjacent to the articular surface, bone cortex, and bone cancellous may respond differently to certain chemical or physical stimuli. However, further research is needed.

There are some articles introducing the beneficial and adverse effects of phenol. Low recurrence rates have been recorded (12, 14, 15, 21), but complications have been less frequently mentioned. Only two articles mentioned complications such as chemical burns and damage to the neurovascular structures and soft tissues nearby (8, 35). It has been suggested that phenol is potentially carcinogenic to other organs after absorption. Thus, after inactivation, the cavity should be thoroughly washed with normal saline to avoid heart, liver, or kidney failure caused by excessive phenol absorption (30, 36). Although, the common use of phenol in practice has guaranteed safety to a certain extent, the other effects on normal tissues with long-term follow-up still require exploration.

##### Anhydrous Ethanol

Anhydrous ethanol (AE) is widely used in microvascular embolization of tumours, tissue fixation, and bacterial killing. The inactivation effect is caused mainly by destruction of the osmotic balance on the cell membrane, which results in dehydration and denaturation of intracellular proteins, lysing of lipids, and finally pyknosis of the nucleus and cytoplasm (37). In the last decade, AE has been widely used for inactivation of the cavity after the removal of GCTB. Compared with phenol, it has superior safety. The usage is similar to that of phenol (Figure 1B). It is generally believed that a better inactivation effect can be achieved by ethanol soaking in the scraped cavity for more than 15 min. However, researchers also found that there were slight differences between different tumour specimens. Considering the particularity of bone tissues (the strong wrapping effect of bone minerals), the inactivation effect is expected to be worse than that of other tumours. Therefore, AE needs to remain in the residual cavity longer to ensure the inactivation effect. This condition can be more easily achieved by soaking.

Oh (38) reported that four (9.5%) patients who had received anhydrous alcohol treatment developed local recurrence, whereas, 15 (48.4%) out of 31 patients who had not received any adjuvant treatment developed recurrence. Lin et al. (35) et al. conducted a prospective study on the inactivation effect of phenol and AE. The results showed that the recurrence rates of the two groups were 12 and 11% within an average follow-up period of 58 months. Short-term complications after the use of AE have not been reported. However, the team of Farah Sharieh, in recent years, indicated that AE inhibits mesenchymal stem cell (MSC) osteochondral lineage differentiation through the activation of forkhead box protein O-specific signalling (39). Thus, we hypothesized that, in the long term, this inactivation method may affect bone repair and osteocompatibility by inhibiting the osteogenetic differentiation or other unknown mechanisms in osteoblast cells. This area of research requires further in-depth mechanistic and long-term clinical prospective studies, which may influence clinicians' choice.

##### Hypertonic Saline

HS has been used to inactivate the residual cavity of GCTB (31). The main mechanism is the formation of osmotic pressure differences inside and outside the cells, which leads to their dehydration. However, due to the particularity of bone tissues, the inactivation range, and cell death rate associated with this method remains to be evaluated. Studies have shown that 20% HS has a positive effect on cell inactivation. Moreover, increasing the temperature or time could significantly increase the inactivation rate. The best inactivation effect could be achieved by increasing the time to more than 30 min. However, the most commonly used treatment in practice is 10% HS for 20 min. Whether the difference in concentration has an effect on the inactivation rate is unknown.

Because of the small number of cases, the exact inactivation effect of HS has not been confirmed. The complications of HS and the effect on bone repair have not been reported. Karjalainen et al. (40) concluded that chondrocytes could tolerate rather high differences in osmolarity, especially in the presence of 20 mM taurine in the cell culture medium, which may partly explain why joint complications were rarely reported and suggest a minor effect on bone repair.

### Physical Inactivation

Compared with chemical reagents, physical inactivation has a greater impact on cells and can theoretically achieve a thorough tumour-killing effect. As a result, it is more commonly used in practice. At present, the most commonly used inactivation methods are high-speed burring, thermal therapy and cryotherapy.

#### High-Speed Burring

In 2002, Ghert et al. (22) first proposed the surgical procedure of high-speed burring (HSB) combined with other treatments after curettage. This idea has been supported by the majority of clinicians. When we remove tumour tissue intraoperatively, the wall of the residual cavity is not actually smooth and is filled with bumpy bone ridges. Therefore, it is difficult to remove tissue between the bone ridges by curettage alone. HSB has a strong cutting effect on bone tissue, which can easily erase the bone ridges to achieve complete removal of tumour tissue and even expand the surgical area. Depths for HSB use have been defined [~1 mm in the normal cortex and 5 mm in the normal cancellous or subchondral bone (41)]. However, it is almost impossible to accurately measure the depth of burring in a surgical procedure. Surgeons can only decide when to end burring according to their own subjective feelings, which may be the reason for the mixed results. Some researchers have argued that the role of HSB in reducing postoperative recurrence is critical (42), while others argue that HSB use after thorough curettage has no effect (2). In contrast, sputtering of fine particles during the use of HSB may lead to the dissemination and implantation of tumour cells (43). However, such a conclusion remains to be confirmed by a large number of studies. Based on a large number of cases, no clinicians have observed that HSB use will increase the possibility of recurrence and invasion, while the convenience to surgeons is obvious. Most researchers contend that HSB alone cannot play a definite role in the reduction of tumour cells and needs to be combined with other inactivation methods (44). Although, there is no literature regarding the effect of HSB on local bone repair, we argue that the mere removal of tissues has very little effect.

#### Thermal Therapy

Thermal cauterization refers to a technique that kills tumour cells by direct or indirect heat shock applied to the wall of the tumour cavity. In recent years, many methods have emerged, including electrotome thermocoagulation (ET) (Figures 1C,D), argon beam coagulation (ABC), high-temperature microwave treatment (HMT), and radiofrequency ablation (RA). Ofluoglu (45) burned the residual cavity with a 120 W ABC combined with 90% phenol inactivation treatment. One case (4%) showed recurrence postoperatively. Lewis et al. (46) burned the residual cavity of 37 cases with an ABC until obvious charring appeared. The power was set at 100 W, and the recurrence rate was 11%. Benevenia et al. (47) reported 93 cases of stage II and III lesions from 1992 to 2007 in which the residual cavity of 33 cases was inactivated with ABC, and the recurrence rate was 15%. Complications, such as postoperative fracture, physeal arrests, synovitis, bursitis, and joint instability, were observed.

**Figure 3:**
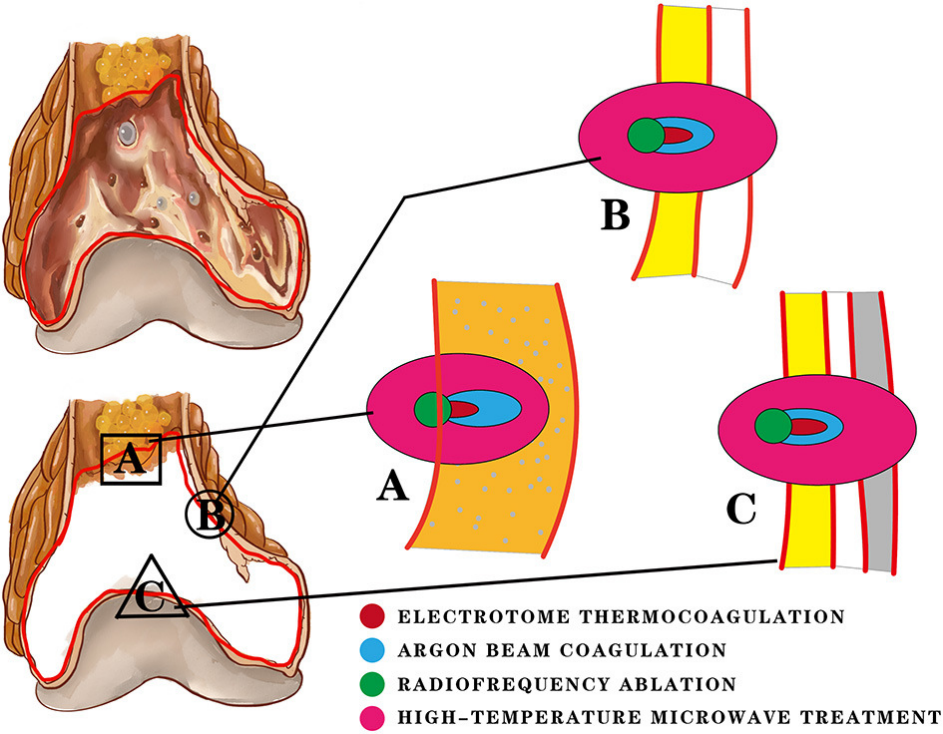
Three locations of the residual cavity after curettage are treated with four thermal therapy methods. The difference in the inactivation range is shown in the figure. **(A)** The orange area represents cancellous bone and bone marrow. Electrotome thermocoagulation (ET) and argon beam coagulation (ABC) are used to burn the tissues at and below their contact point, producing a certain depth of inactivation (red). ABC provides a continuous current and has a deeper distance (blue) of inactivation than ET. Radiofrequency ablation (RA) and high-temperature microwave treatment (HMT) both have scope effects. RA depends on heat conduction and moisture, which results in a small inactivation range (green). Most of the heat is concentrated in the residual cavity. HMT is not limited by moisture and heat conduction, so it has a large inactivation range. **(B)** The yellow area represents the residual cancellous bone; the white area represents the bone cortex. Due to the poor thermal conductivity and low water content of the bone cortex, the inactivation distance of ET is usually limited within the cancellous bone and cannot break through the bone cortex. RA produces only a small inactivation range. The inactivation distance of ABC can break through the cortex slightly. HMT is not limited by the cortex and can cause a wide range of inactivation, causing damage to extra-skeletal tissues. **(C)** The gray area represents the cartilaginous surface of the joint. ET and RA have no effect on the bone cortex and cartilage surface based on the above description. The thermal effect of ABC is limited to the cortex. However, the unrestricted inactivation range of HMT is likely to cause irreversible articular surface damage and some joint complications.

Different technologies rely on different heat production mechanisms. ET produces heat mainly by high-frequency and high-voltage current at the electrode tip. ABC can continuously transmit the coagulation current to the surroundings of the electric tip, producing a better thermal effect. RA produces a resistance electrothermal effect in tissues through electrode catheters. Microwave energy is generated by the rotation and continuously accelerated collision of dipoles in the microwave field. RA is easily affected by the concentration of free ions and the electrical conductivity. The expansion of the solidification range relies mainly on heat conduction and dissipation. Therefore, its application is limited due to the low water content and poor thermal conductivity of bone tissue. It is often applied to liver, kidney, and other soft tissue tumours. For microwave energy, the dipoles in the magnetic field are all heat sources. The concentration of free ions and conductivity have little influence. Thus, the surrounding tissues can acquire higher thermal effects. RA and HMT have scope effects rather than point effects (Figure 3). If we cannot control the high temperature range, damage to surrounding tissues and other serious complications may occur. As a result, these two methods are less commonly used. Regardless of the mode of heat production, the fundamental purpose of heat burning is to cause proteins inside and outside the cell to coagulate and denature, leading to rapid tissue necrosis. In that way, what degree of heat can cause necrosis of bone tumour tissue?

Moritz and Henriques proposed the principle that high temperatures should be used for a short period and low temperatures for a long period. However, no specific temperature was proposed. In 1986, Nelson et al. (48) reported that the temperature and time of osteonecrosis were 50°C for 6 min. Subsequently, studies on the correlation between heat conduction and heat apoptosis were conducted. Heck et al. (49) used ABC at 50, 100, and 150 to cauterize the cancellous bone of pigs and achieved necrosis depths of 1.0 ± 0.5 mm, 2.9 ± 1.0 mm, and 4.2 ± 0.7 mm, respectively, indicating that the necrosis depth would increase correspondingly with increased power. However, there was a study in China proposing that although, the heat and radiation distance were proportional to the time and power, the inactivation distance tended to be stable. Thus, we hypothesized that inactivated tissues that have undergone rapid coagulation under ultrahigh temperature may form a strong “protective shield,” thus preventing further damage to other cells. Phimolsarnti et al. (50) conducted an *in vitro* study on the temperature and apoptosis rate in 2012. The results were consistent with the findings of Li Leibo. The apoptosis rate of cells was proportional to the temperature and time. However, when the temperature was set to 50°C, the apoptosis rate did not increase with increasing time but tended to be stable or even decreased. Moreover, Rapin also found that tumour cells could maintain their growth and survival better than normal cells. However, after thermoablation between 47 and 50°C for periods of 20 and 30 min, chondrocytes and osteoblasts had lower apoptosis rates than giant cell tumours, which is also the current concern of surgeons regarding which temperature can ensure effective tumour killing and activity of normal tissues simultaneously. Rapin proposed that 47°C for 20 to 30 min is the most suitable choice for GCTB. However, many questions remain concerning this temperature because the temperature used in the clinic is much higher. The heat used in practice may cause serious damage to normal tissues during tumour killing.

Subsequently, local bone repair becomes important after injury. Bone morphogenetic protein (BMP), vascular endothelial growth factor (VEGF), transforming growth factor B (TGF2B), and other factors participate in repair. What about the heat tolerance of these factors? Most attention has been paid to BMP, which has strong heat resistance. Within a certain temperature range, its activity gradually increases with increasing temperature. After heat treatment at 70°C for 30 min, its activity reaches a maximum. Then, as the temperature increases, the activity gradually decreases. Bone induction activity still occurs after heating at 170°C for 10 min and 140°C for 30 min (51). Lucas et al. (52) studied a chemokine with a molecular weight between 50,000 and 90,000. The role of this factor is to guide the movement of MSCs, which eventually differentiate into osteoblasts and osteoclasts. The results showed that the heat resistance of this factor was strong. It could be inactivated by heating at 100°C for 10 min. It was suggested that 50°C could be used as a dividing line in terms of the activity change of biologically active factors. When the temperature reached 50°C, the antigenicity began to weaken and then gradually disappeared above 70°C (such as alkaline phosphatase). These studies suggest that bone-induced activity still exists under acute thermal stress, but it depends on temperature and time to maintain good activity (53). In summary, 50–65°C for 30 min can achieve an inactivation effect and confirm bone repair activity. The temperature in practice is often too high (>100°C), and local bone repair is likely to be inhibited to varying degrees. Some researchers have mentioned this complication after inactivation with ABC. Bone repair usually begins immediately when bone defects form. However, reconstruction of the local blood supply often requires a longer time. Only by one year did the inactivated segments recover close to the normal lever. Inhibition of VEGF and its products by hyperthermia (54) may explain the long vascular remodelling period. These observations also indicate that the initial bone repair of the residual cavity often involves reactive hyperplasia and crawling replacement of surrounding bone tissues. Reconstruction of the local osteogenic microenvironment often takes more than one year. It is advised that patients undergo longer periods of internal fixation and proper immobilization to match the lengthy process. There have been many studies on the effects of hyperthermia on the peripheral blood supply. The results showed that a prolonged heating time and increased temperature can lead to the stasis of vessels (55–59). However, in practice, the bone cortex blocks most of the heat due to its poor thermal conductivity. With proper protection, hyperthermia is less likely to cause complications of peripheral vessels.

At present, the application of heat inactivation in the field of bone tumours is becoming increasingly frequent. This method can not only reliably reduce the recurrence rate but also ensure fewer clinical complications. However, many questions remain regarding the choice of temperature, time and power. More attention and *in vitro* and *in vivo* experiments are needed.

#### Cryotherapy

**Figure 4:**
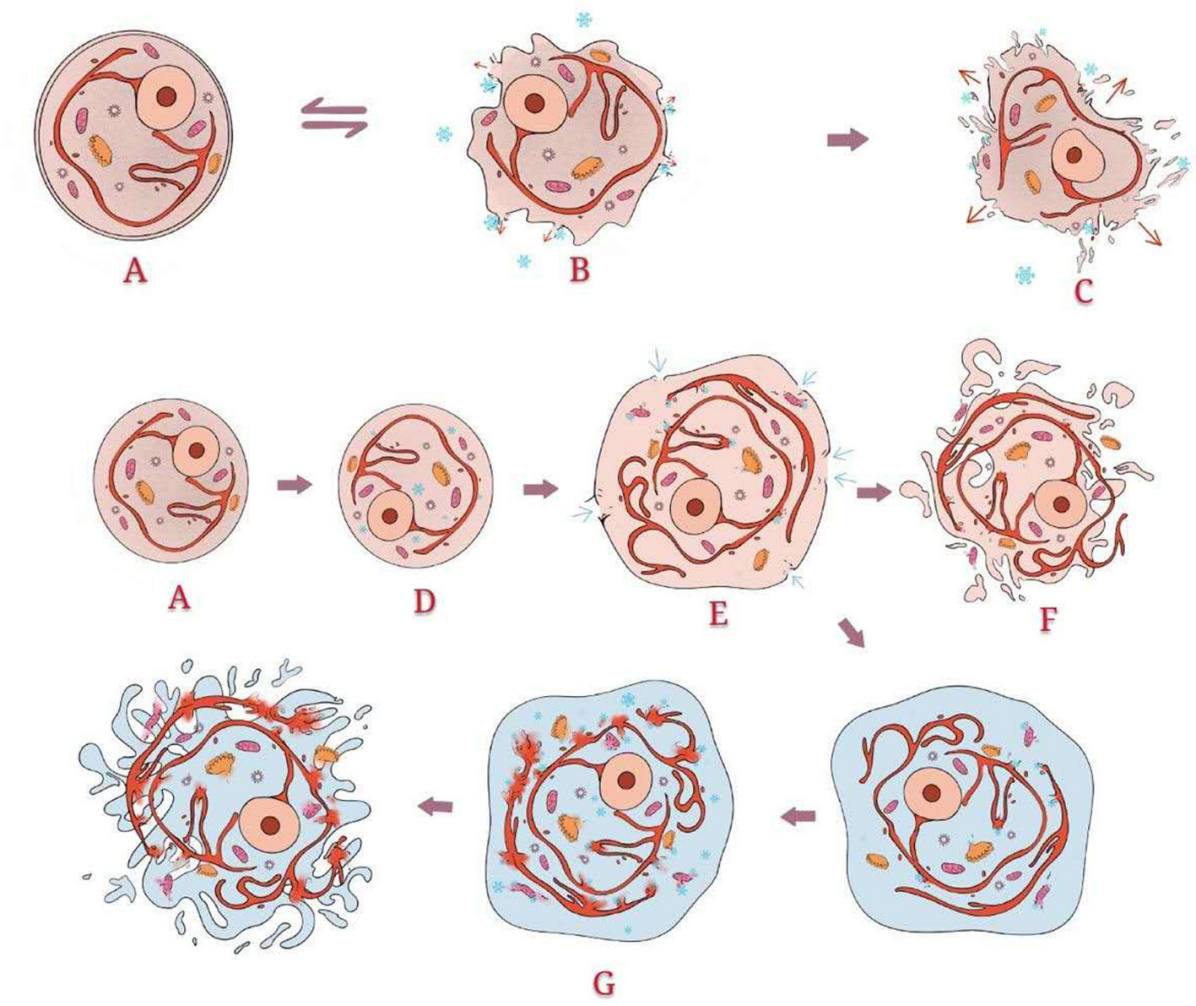
Direct damage: Normal structure of the cell. When the temperature drops slowly and does not reach −40°C, the effect of hypothermia occurs mainly outside the cells. Ice crystals form outside the cells, which directly destroys the cell membrane and changes the osmotic pressure. Intracellular fluid flows to the extracellular area along the osmotic pressure gradient, leading to shrinkage and even death of the cells. However, this process is partially reversible. When the temperature drops sharply and reaches below −40°C, ice crystals form inside the cells, directly destroying the internal organelles. Simultaneously, extracellular fluid flows to the intracellular area, resulting in cell swelling. Through thawing, the swelling of cells becomes severe. Some cells split into fragments. The swelling cells without splitting enter the second freeze-thaw cycle. As the present intracellular fluid is more abundant than that in the first cycle, more ice crystals form. The cells finally split due to the greater osmotic pressure difference.

**Figure 5:**
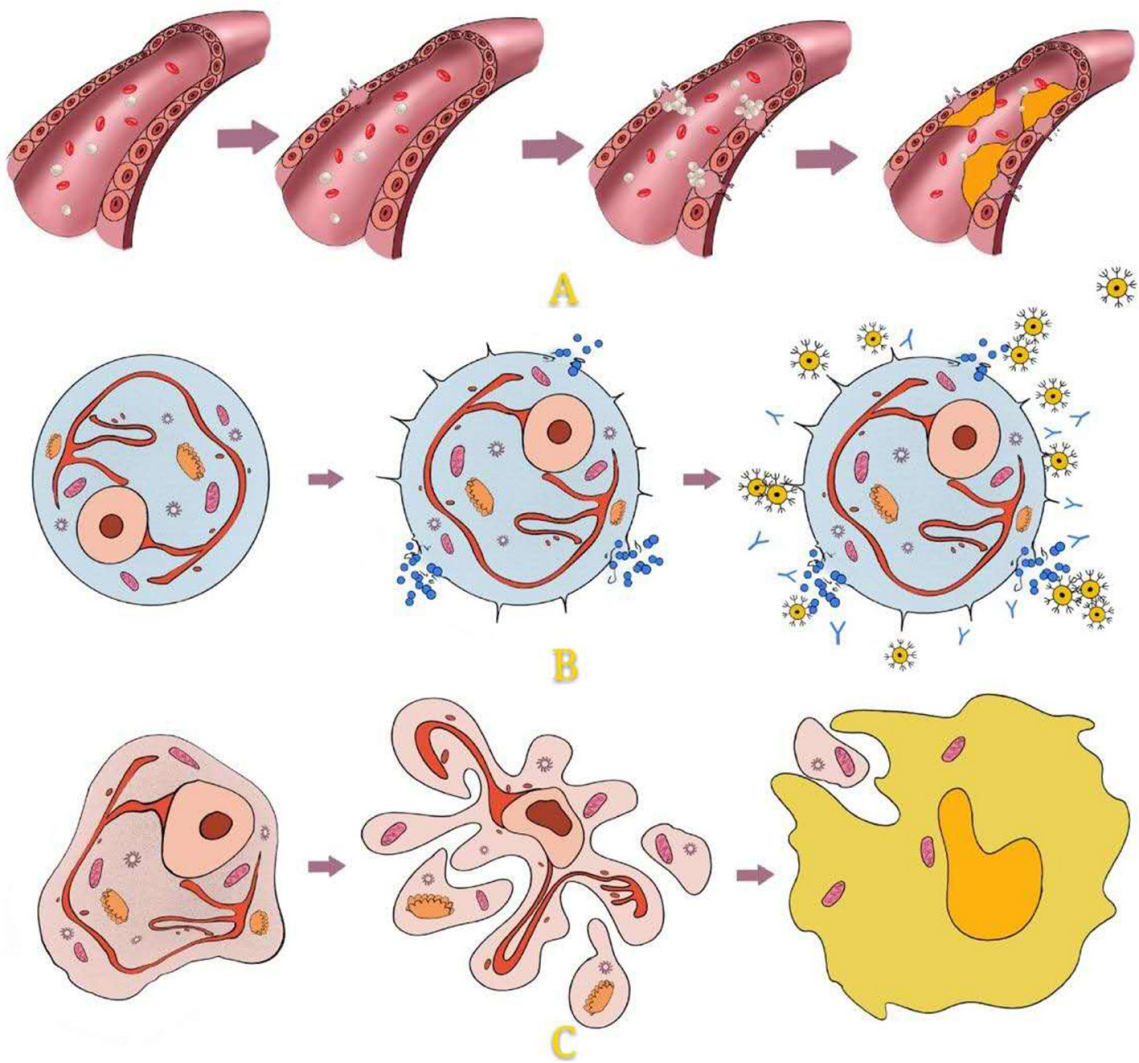
Indirect damage: For peripheral areas that are not in direct contact with hypothermia, temperature cannot cause direct cell disruption. This kind of cell damage is caused mainly by injury to vascular endothelial cells, immune responses, and apoptosis. Damage to vascular endothelial cells leads to the aggregation of platelets, thrombus formation, stasis of blood, and local ischaemia, finally causing cell damage. Hypothermia causes membrane damage and then antigen exposure, which causes the production and accumulation of antibodies and cytotoxic T cells. Hypothermia activates the expression of apoptosis genes (proteocleavage-activated caspases 3, 8, and 9, Bax, Bcl2, etc.), which leads to the release of enzymes and the formation of apoptotic bodies. Apoptotic bodies are internalized by phagocytes.

After hyperthermia was found to be useful for inactivation, the destructive effect of hypothermia on cells was considered. The earliest hypothermia therapy can be traced back to the 19th century. With the development and utilization of resources, the temperature that could be achieved gradually decreased until the appearance of liquid nitrogen, which marks the beginning of modern cryotherapy and offers hypothermia at −196°C (60, 61). The mechanism of cell disintegration caused by hypothermia is complex, including direct, and indirect damage. Among them, direct damage includes two main damage processes: quick freezing and slow melting (Figure 4). Indirect damage involves circulation failure, immune clearance, and apoptosis (62–65) (Figure 5). Compared with healthy cells, tumour cells have greater and variable resistance to cold (66). Therefore, the freezing speed, application time, and frequency of freeze-thaw cycles usually increase to achieve a better tumour-killing effect (67). At present, −50 to 70°C is considered an effective temperature (68). The use of at least two freeze-thaw cycles with fast freezing and slow thawing is particularly effective (69).

Marcove et al. (70–72), Marcove and Miller (73) first applied cryotherapy to bone lesions to relieve pain. Cryotherapy is widely recognized as having a significant effect on pain relief (74–76). In 1978, Marcove (77) treated 52 patients with GCTs by large incision, full reveal, thorough curettage, and repeated freezing to −20°C. The overall recurrence rate was 23%, which is lower than that of simple curettage [40–55% (78, 79)]. This process provided an effective tumour clearance method for surgeons. Subsequent reports have shown that curettage combined with cryotherapy can significantly reduce the recurrence rate (68, 80–82). Concurrently, many complications have also been reported, including postoperative fracture, skin necrosis, transient nerve palsy, infection, traumatic arthritis, tumour malignancy, and bone graft non-union. The incidence has ranged from 12 to 50% (83, 84). Moreover, individual cases showed that cryotherapy resulted in systemic cytokine-mediated cryo-shock syndrome with hypotension, dyspnoea and disseminated intravasal coagulation (85). Various studies have also shown that the safety of cryotherapy is not guaranteed.

Rapid freezing and slow thawing for 3 cycles has been reported to increase the surgical boundary to 2 cm (9, 77). The necrotic distance increased correspondingly with the decrease in temperature (86). Unlike the extensive damage caused by hyperthermia, the inactivation rate of cryotherapy is relatively low. Cells in direct contact often suffer acute damage, while others depend on delayed immune responses and apoptotic mechanisms. The necrosis of the microarterial system after cryotherapy is often difficult to reverse. However, the large vessels and nerves are generally not damaged. Even if they are damaged, recovery commonly occurs within 24 h (86–88), which ensures the basic safety and wide application of cryotherapy. In the initial stage of cryotherapy, direct infusion using liquid nitrogen was the most commonly used technique. However, it greatly increased the incidence of complications. Later, with the continuous improvement of technology, the plug-in type, pressor spray type, and a modern argon-helium knife were applied. An argon-helium knife uses argon for freezing and helium for thawing. Although, the tumour-killing effect is excellent, it is rarely used in practice due to its high price (7). In cryotherapy, temperature control and detection systems are also important. Researchers have conducted numerous studies in this area (89–93), which are not further discussed herein.

For osseous lesions, cryotherapy offers some unique advantages over other adjuvant treatments since it will kill cells but leave the inorganic osseous framework intact, which can remain as a matrix to achieve better bone healing (82). Generally, bone healing is achieved 3 months after cryotherapy, which marks the beginning of weight bearing for patients (94). At present, there is a lack of research examining the effects of 68 temperatures (below −50°C) on the activity of osteogenesis-related cytokines (BMP, VEGF, etc.,) and the expression of osteogenic genes (Runx2, OSX, etc.,). Therefore, information about bone repair of the residual cavity after cryotherapy remains sparse.

The combined application of cryotherapy has recently become a trend. Because liquid nitrogen is difficult to control, researchers created a semisolid cryogen, a freezing nitrogen ethanol composite, which achieved good security, and a low recurrence rate. The freshly prepared freezing nitrogen ethanol composite was frozen to −136°C. When it was applied in the cavity, an isotherm of −122°C across a piece of 10 ± 0.50-mm-thick bone was achieved after several minutes (95). The combination of cryotherapy and immunoregulatory drugs has also been reported. In a mouse model of melanomas, it was shown that the combination of cryotherapy and CpG-B oligodeoxynucleotides resulted in significantly more effective tumour control than monotherapy (96), providing us with new ideas for the treatment of GCTB. The combination of cryotherapy with immunomodulators may also be effective in reducing the recurrence and metastasis of GCTB. However, there are many kinds of immunomodulators with obvious side effects in the clinic. To achieve a reliable conclusion, the pros and cons of many studies must be evaluated.

### Cavity Filling and Osteogenic Induction

After completion of tumour cell reduction, cavity filling, and osteogenic induction are also necessary. The filling materials of the residual cavity include autogenous bone, allograft bone (AB), and synthetic bone substitute materials (SBSMs). Reconstruction using autogenous bone is usually performed with a segment of the fibular or iliac wing. However, due to the irregularity of the residual cavity and the fixed shape of the autogenous bone, it is often difficult to achieve complete filling and good mechanical results using autogenous bone alone. Thus, AB and SBSM are widely used. AB is excellent as an implant material. Its structure and properties are completely consistent with the bone tissues surrounding the cavity. Pogrel et al. (86) assessed the influence of bone grafts on bone healing in the mandible of minipigs and showed that the group with bone grafts achieved a better clinical healing effect while that without bone grafts showed a 50% rate of wound breakdown and sequestrum formation with delayed healing. However, bone grafting does not appear to increase the bone density or rate of bone formation in defects at three months postoperatively, which suggests a minimal effect of bone grafts on bone repair and a long duration of the bone repair procedure. When AB is used as the only implant, it cannot provide sufficient local mechanical strength in the early stage. With the continuous development of materials, an increasing number of SBSMs have been applied, including apatite-wollastonite-containing glass ceramics (AWGCs), hydroxyapatite (HA), tricalcium phosphate (TCP), and PMMA. The ideal SBSM should have good biocompatibility and biodegradability. Simultaneously, it should promote the formation of new bone tissues. Excluding PMMA, the other three materials are all biocompatible, bioactive, and osteoconductive (97–100). The unique point of PMMA is its good mechanical strength and deformability. It has also been reported to reduce local recurrence due to its cytotoxic effect and heat product ability (22, 101, 102). However, its disadvantages are also obvious. Due to the difference between PMMA and surrounding tissues in nature, stress-induced loosening (103), peripheral fractures, accelerated loss of articular cartilage, and displacement of the bone cement block are common complications. Therefore, internal fixation is advised (104) when using PMMA as the filling material. TCP is a bone substitute material that is widely studied and applied worldwide. It has ideal biocompatibility and biodegradability because of its similar physicochemical properties to bone tissues. However, TCP is granular and difficult to shape. It is often necessary to combine with other biological excipients (such as cross-linked gelatine) to achieve a better adhesion effect.

At present, the application of composite materials has become a trend. The combination of different inorganic materials, inorganic and organic, inorganic materials, and bone induction factors as well as organic materials and bone induction factors has gradually appeared. Kotrych et al. (105) reported that the application of Highly Injectable Bi-Phasic Bone Substitute (CERAMENT) achieved good clinical effects. Hattori et al. (106) found that HA/TCP was more bioresorbable and osteoconductive than HA or AWGC. Shin et al. (107) presented successful reconstruction with autogenous bone grafts and autologous bone marrow mesenchymal stem cells. Other composites have also been described, including PL GA capsules containing RHBMP-2 (108) and poly(DL-lactic-co-glycolic acid)/calcium-phosphate cement composites (109). In the next phase, the selection and production of bioactive substances will be more systematic and efficient, and the proportion of biomaterials in composites will be more specialized. Accompanied by the progress of the controlled-release system, the promotion of local bone repair and bone fusion by filling materials will become a kind of periodic treatment.

## Discussion

We listed the postoperative recurrence rates and complications caused by different management strategies for the residual cavities of GCTB in the last ten years (12, 16, 20, 35, 47, 104, 110–116) (Table 1). The recurrence rates and incidences of complications vary between different treatments. Even when using the same method, they can also be different, possibly because the application processes are different. In general, a definite pattern or rule is lacking in the management of the residual cavity to guide the choice of surgeons and ensure good clinical results. We summarize the characteristics of each method (Table 2) and propose some of our own opinions. For HS and AE, we believe that the inactivation effect of soaking is far superior to that of smearing. For phenol, smearing is relatively safe due to its high biological toxicity. The onset time and frequency should be guaranteed. When inactivating the residual cavity by hyperthermia, the wall should be burnt inch by inch to ensure that every corner is covered. We do not recommend using an exceedingly high power over a long time. The total cauterization time should be less than 15 min. Direct dumping of liquid nitrogen in cryotherapy is an approach with which we strongly disagree. Use of the Plug-in Type and the Pressor Spray Type can better ensure safety.

**Table 1:** Comparison of different methods for residual cavity management in the last 10 years.

**Anthor**	**Year**	**Patients**	**Procedure**	**Recurrence**	**Complication**
Errani C (20)	2010	64	Phenol + alcohol + cement	12.5%	None
Pietschmann MF (110)	2010	34	Phenol + bone graft/cement	32.4%	None
		13	Bone graft + cement	53.9%	None
Gaston CL (16)	2011	84	HSB ^*^ + cement	14.3%	Osteoarthritis (7.3%) Neuroma (1.2%) Fracture (4.8%) Deep infection (2.6%)
		246	HSB ^*^	29.7%	Ulnar abutment (1.2%) Neuroma (0.4%) Fracture (1.6%) Painful cavity (0.8%) Deep infection (0.4%)
Klenke FM (12)	2011	40	Phenol + PMMA ^*^	14.6%	None
		32	Phenol + bone graft	34.4%	None
Lin WH (35)	2011	26	HSB ^*^ + phenol + cement	12.0%	Narrowing of the joint space (3.8%)
		35	HSB ^*^ + 95% ethanol + cement	11.0%	None
Benevenia J (47)	2012	39	Phenol + PMMA ^*^ /bone graft	17.9%	Physeal arrests (5.1%) Synovitis (2.6%) Arthrofibrosis (2.6%) Bursitis (2.6%) Deep infection (2.6%)
		54	ABC ^*^ + PMMA ^*^ /bone graft	16.6%	Postoperative fracture (3.7%) Physeal arrests (1.85%) Synovitis (1.85%) Bursitis (1.85%) Joint instability (1.85%)
Moon MS (111)	2013	23	EC ^*^ + HSB ^*^ + phenol + cement	None	None
Gao ZH (112)	2014	34	HSB ^*^ + phenol + bone graft	35.3%	None
		31	HSB ^*^ + phenol + cement	12.9%	None
van der Heijden L (113)	2014	82	Phenol + PMMA ^*^	28.0%	Osteoarthritis (7.3%) Infection (1.2%) Nonunion (2.4%)
		26	Liquid nitrogen + PMMA ^*^	31.0%	Osteoarthritis (15.4%) Infection (3.8%) Fracture (3.8%) PMMA Leakage (3.8%)
		24	Liquid nitrogen + bone graft	38.0%	Osteoarthritis (12.5%) Infection (8.3%) Fracture (8.3%) Nerve palsy (4.2%)
Benevenia J (114)	2017	21	ABC ^*^ + hydrogen peroxide ^±*^ phenol + allograft ^±*^ PMMA ^*^	29.0%	Osteoarthritis (4.8%) Periarticular fracture (4.8%)
		22	ABC ^*^+ hydrogen peroxide ^±*^ phenol + PMMA ^*^	32.0%	Osteoarthritis (31.8%) Periarticular fracture (22.7%)
Takeuchi A (103)	2018	26	Phenol + CPC ^*^	11.5%	Osteoarthritis (3.8%) Chronic synovitis(3.8%) Fracture (3.8%)
Sirin E (115)	2020	79	EC ^*^+ PMMA ^*^	5.1%	Infection (2.5%)
Ke J (116)	2021	54	Microwave + bone graft + cement	3.7%	Infection (1.85%) Osteoarthritis (3.7%) Wrist joint subluxation (1.85%) Fracture (1.85%)

* *HSB, High Speed Burring; ABC, Argon Beam Coagulation; PMMA, Polymethyl Methacrylate; CPC, Calcium-Phosphate Cement; EC, Electrocauterization*.

* *The amount of these literatures did not represent the literature volume of the whole review*.

*± *: with or without*.

**Table 2:** Summary of various inactivation methods.

**Method**	**Usage**	**Action time**	**Temperature**	**Range**	**Efficiency**	**Side effects**
Phenol	Smear	6 min ^*^ 3 times	No heating	Small	Medium	Absorptive toxicity Carcinogenicity damage to vessels and nerves
Absolute ethanol	Soak	15 min	No heating	Small	Medium	/
Hypertonic saline	Soak	30 min	Heatable	Small	Low	/
Electric knife	Point cauterization	Long	Low	Medium	High	/
ABC	Point cauterization	Short	High	Large	High	Postoperative fracture Physeal arrests Synovitis Bursitis
Liquid nitrogen	Range freezing	At least 2 cycles	−50~-70°C	Large	Medium	Postoperative fracture Skin necrosis Infection Transient nerve palsy Traumatic arthntis Bone graft non-union Cryo-shock syndrome

* *Range: inactivation range*.

*ABC, Argon Beam Coagulation*.

In comparisons of phenol and ethanol, phenol, and ABC, and phenol and liquid nitrogen (Table 1), several researchers concluded that there was no difference in postoperative recurrence. Suggestions for surgeons were based on the incidence of complications. Accordingly, phenol and liquid nitrogen are not recommended. The inactivation effect of HS is weak, and it requires a long time to have an effect. Although, its safety is extremely high compared with phenol and ethanol, we do not generally recommend the use of HS for residual cavity treatment. Ethanol is similar to phenol in all aspects but is much safer. Thus, we recommend the use of ethanol for inactivation. Hyperthermia is a safe and efficient inactivation method. Among approaches, ET and ABC are recommended for use. However, as we stated previously, excessive temperatures are not recommended. Complications following high-power ABC inactivation have been reported. Therefore, we recommend the use of ET or low-power ABC for treatment. We also propose a scenario in which the combination of ethanol and hyperthermia can achieve better results. We believe that after ethanol immersion and washing, a low concentration of ethanol will remain in the tissues. At this point, when we conduct hyperthermia, deeper depths of inactivation may be achieved without increasing the temperature. This idea has not been confirmed, but it could open up a new avenue for surgeons. Of course, complete removal of the tumour is of the utmost importance before any inactivation. When this cannot be ensured with curettes alone, use of HSB is also particularly important. PMMA is recommended by most researchers as the filling material. Re-inactivation of PMMA is beneficial for management of the residual cavity, but complications are also possible. We believe that allograft implantation is also a good option. Patients with bone grafts have less discomfort and fewer complications after surgery. With adequate internal fixation, mechanical strength during the recovery period can also be partially assured. Moreover, after combination with other osteo-inductive materials, composite bone grafts are the best choice.

The main foothold of this review is the implementation of extended curettage through various inactivation methods, which were described and compared in detail. However, most of the conclusions we have mentioned above are not feasible for GCTB that has penetrated the articular surface and surrounding soft tissues. For extra-articular and cortical GCTB, we should first evaluate the possibility of curettage. When articular surface destruction exceeds 50% or large areas of soft tissues are affected, intralesional curettage is not recommended (117). At this point, we should choose segmental resection to ensure low postoperative recurrence. For GCTB with a small range of invasion to the articular surface and soft tissues or with pathological fracture, we should pay attention to the following points when we choose to perform curettage. The use of chemical reagents can only involve smearing rather than soaking. Soaking may lead to leakage of fluid into the joint cavity and surrounding tissues, which will increase the incidence of complications. Use of liquid nitrogen is not permitted. Hyperthermia is still an alternative method. However, it should be noted that the power and time when used at juxta-articular points should be appropriately reduced to avoid large-scale destruction of articular chondrocytes and synovial tissues. In general, the effect of tumour reduction under these conditions was greatly reduced, and the possibility of recurrence increased. Moreover, filling materials may also have a certain effect on joint function. Therefore, for these special GCTBs, the choice of surgery should be carefully decided after communication with patients.

In 2013, a humanized monoclonal antibody against the receptor activator of nuclear factor-κB-ligand (RANKL), denosumab, was approved for the treatment of advanced GCTB. Because this review does not focus on drug management, we do not elaborate on the mechanism of denosumab. At present, various disadvantages of denosumab have been reported by researchers (118, 119). Therefore, we do not support the use of denosumab in combination with various residual cavity management strategies. Advanced technology has rarely been applied in this field, including nanocryotherapy (120), controlled and sustained release systems, microsphere carriers, and some new osteo-induction and reconstruction materials. The addition of these new technologies will achieve accurate tumour-killing effects with reduced loss of normal tissues. Moreover, the promotion of bone repair will also become an important part of adjuvant therapy.

## Conclusion

Compared to curettage alone, management of the residual cavity can effectively reduce the recurrence of giant cell tumours of bone. It is a complex and multidisciplinary process that includes three steps: local control, cavity filling, and osteogenic induction. In terms of local control, HSB can enlarge the area of curettage but may cause the spread and planting of tumour tissues. Among the inactivation methods, AE and hyperthermia therapy are relatively safe and efficient. The combination of the two may achieve a better inactivation effect. When inactivating the cavity, we need to adjust the approach according to the invasion of the tumour. Filling materials and bone repair should also be considered in management.

## Author Contributions

YW conceived and wrote most of the manuscript. CW made the figures and tables. QT and JL collected the related data. HL provided partial financial support. YF thoroughly revised and amended the manuscript. All authors contributed to the article and approved the submitted version.

## Conflict of Interest

The authors declare that the research was conducted in the absence of any commercial or financial relationships that could be construed as a potential conflict of interest.

## Publisher's Note

All claims expressed in this article are solely those of the authors and do not necessarily represent those of their affiliated organizations, or those of the publisher, the editors and the reviewers. Any product that may be evaluated in this article, or claim that may be made by its manufacturer, is not guaranteed or endorsed by the publisher.
